# Pure 46, XY gonadal dysgenesis and 46, XY complete androgen insensitivity syndrome: A case report

**DOI:** 10.1097/MD.0000000000038297

**Published:** 2024-06-21

**Authors:** Tengge Yu, Li Liu

**Affiliations:** a Department of Gynecology and Obstetrics, West China Xiamen Hospital of Sichuan University, Xiamen, China; b Department of Emergency, The First Affiliated Hospital of Jiamusi University, Jiamusi, Heilongjiang, China.

**Keywords:** 46XY female, androgen insensitivity syndrome, germ cell tumor, gonadal dysgenesis, gonadectomy, long term hormone replacement therapy, sexual differentiation

## Abstract

**Background:**

Disorders of sex development (DSD) are congenital conditions characterized by atypical development of chromosomal, gonadal, and phenotypic sex. 46, XY DSD can result from disorders of testicular development or androgen synthesis.

**Methods:**

We present 2 rare cases of 46, XY DSD, specifically XY pure gonadal dysgenesis and complete androgen insensitivity syndrome.

**Results:**

Both cases underwent prophylactic gonadectomy due to the elevated risk of gonadal malignancy. Bilateral gonadoblastoma and dysgerminoma were diagnosed on one side, while Leydig cell hyperplasia and only Sertoli cells were diagnosed in the seminiferous tubules on both sides. The normal menstruation for the pure gonadal dysgenesis patient only as CAIS patients never menstruate. Estrogen replacement therapy was administered periodically to promote the development of secondary sexual characteristics and menstruation in pure gonadal dysgenesis case, as well as to prevent osteoporosis. Follow-up examinations revealed no tumor recurrence, and the patient with Swyer syndrome had regular menstrual cycles.

**Conclusion:**

Laparoscopic bilateral prophylactic gonadectomy and long-term hormone therapy with patient counseling and support are recommended.

## 1. Background

A disorder of sex development (DSD) is defined as a congenital condition in which the development of chromosomal, gonadal, or anatomical sex is atypical.^[1–3]^ According to the Chicago classification, DSD is subdivided into 3 groups based on cytogenetic, hormonal, gonadal histology, and clinical findings: 46, XY (primary disorder of testicular development or disorders in androgen synthesis or action), 46, XX (disorders of ovarian development, congenital adrenal hyperplasia, aromatase deficiency), and sex chromosome disorders (Klinefelter syndrome, Turner syndrome, chimerism, mixed gonadal dysgenesis).^[1,3,4]^

The overall rate of individuals with Y chromosome material is approximately 1 in 20,000 births. 46, XY DSD may occur at any point in the sexual differentiation pathway, which is a complex process involving gene interactions, androgen synthesis, and hormone regulation through the interaction of ligands with their corresponding receptors.^[5]^ The diagnosis of patients with 46, XY DSD is primarily clinical and is typically identified during investigations for primary amenorrhea or delayed puberty. The most common symptom that prompts patients to consult a physician is primary amenorrhea. This disorder is defined as either a lack of menarche by 14 years of age in the absence of secondary sexual characteristics or a lack of menses by 15 years of age in the presence of normal growth and secondary sexual characteristics.^[6–8]^

We present 2 uncommon cases of 46, XY DSD patients who underwent prophylactic gonadectomy due to the risk of developing gonadal malignancy. One case involves XY pure gonadal dysgenesis, which is associated with impaired testicular development, while the other case involves complete androgen insensitivity syndrome (CAIS), characterized by the inability of the androgen receptor to respond to androgens, despite normal testicular development and androgen synthesis. The normal menstruation for the PGD patient only as CAIS patients never menstruate. We also discuss the pathophysiological processes and the optimal timing of gonadectomy, along with the evidence on the risk of neoplasia in patients with 46, XY DSD.

## 2. Case presentation

### 2.1. Case 1

**Figure 1.**
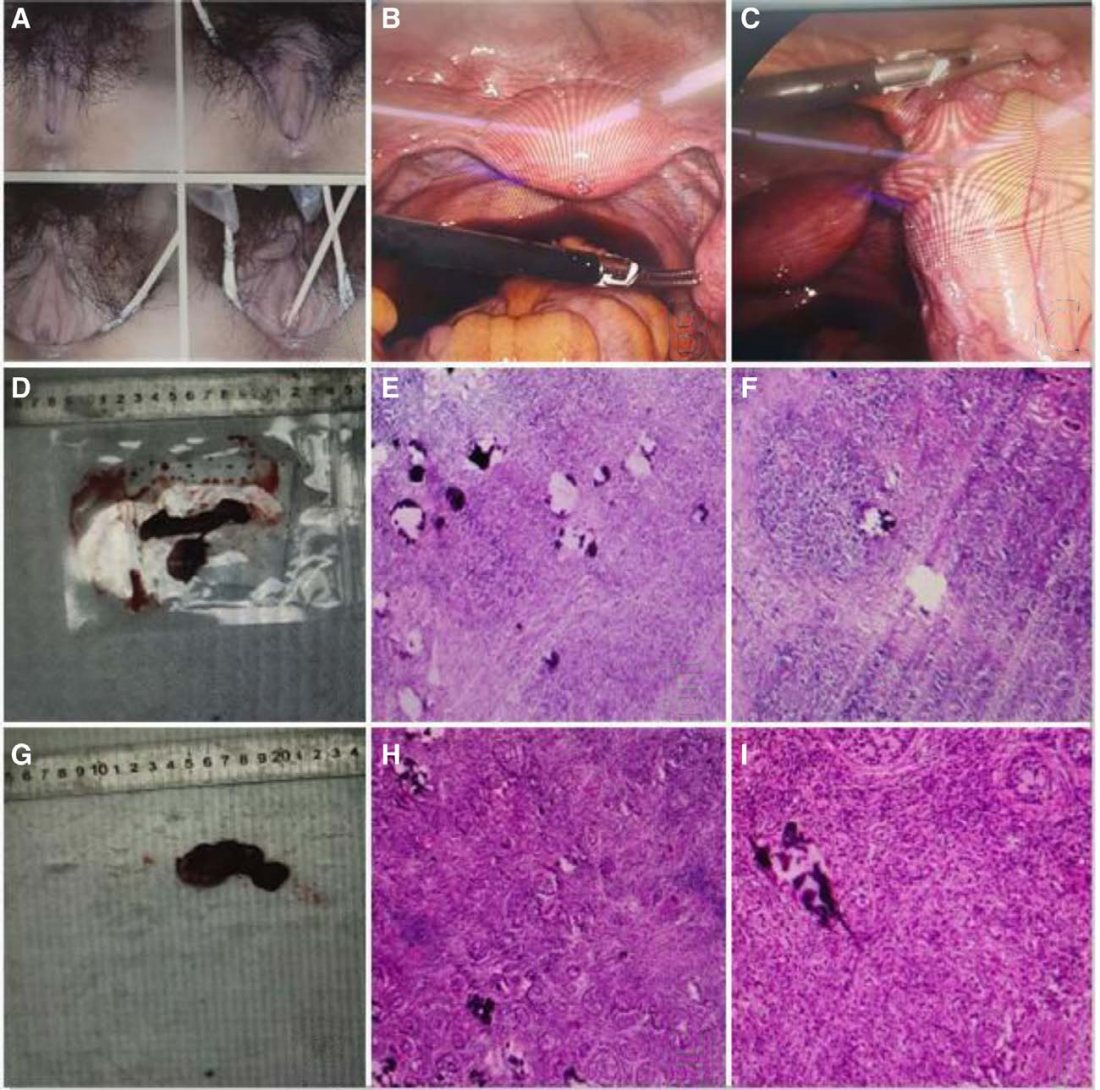
Clinical findings of a patient with XY pure gonadal dysgenesis. (A) Normal external female genitalia with an intact hymen. (B) Small uterus and left streak ovary and fallopian tube. (C) Right streak ovary and fallopian tube. (D) Gross finding of left inguinal testes in a patient with XY pure gonadal dysgenesis. (E, F) Microscopic finding of a left streak ovary in a patient with XY pure gonadal dysgenesis (gonadoblastoma). The tumor cells, round or ovoid in shape, form nests that vary greatly in size. The nests are surrounded by fibrous connective tissue and have distinct borders. At the center of the nest are large and round germ cells with abundant transparent cytoplasm and deeply stained nucleus. The supporting cells and granular cells, small in size and spindle or ovoid in shape, are arranged in clusters around the nests. (G) Gross finding of right inguinal testes in a patient with XY pure gonadal dysgenesis. (H, I) Microscopic finding of a left streak ovary in a patient with XY pure gonadal dysgenesis (dysgerminoma). The tumor cells are large in size and round or ovoid in shape, and have distinct borders. The nucleus at the center of the cell is large and round, and nuclear division is often observed. There is abundant transparent cytoplasm. Lymphocyte infiltration is observed in the connective tissue.

A 16-year-old girl was referred to our clinic for an evaluation of primary amenorrhea and delayed puberty. She had no pertinent medical or family history. Her weight was 50 kg, and her height was 166 cm, and the body mass index was 18.1 kg/m^2^. The patient had underdeveloped breasts (Tanner breast stage 2) and minimal pubic hair growth (Tanner pubic hair stage 2). Examination of the genitalia revealed that the patient had normal female external genitalia with an intact hymen and a 4 cm deep vagina (Fig. 1A). Transabdominal ultrasonography and magnetic resonance imaging (MRI) revealed a uterus measuring approximately 31 × 42 × 40 mm; however, neither ovaries nor testes were visible. Laboratory evaluation revealed elevated levels of follicle-stimulating hormone (25.98 IU/L) and very low estradiol levels (<5 pg/mL). The serum level of luteinizing hormone was 20.41 IU/L, and the testosterone level was 2 ng/dL, both of which were within the normal range for women of that age. The serum level of anti-Müllerian hormone (AMH) was 0.93 ng/mL. A karyotype was obtained, which showed a normal male karyotype of 46, XY. We planned to perform prophylactic laparoscopic gonadectomy based on the diagnosis of XY gonadal dysgenesis. Laparoscopic exploration revealed the presence of bilateral streak gonads with a small uterus and normal-appearing fallopian tubes (Fig. 1B–D and G). The patient underwent laparoscopic bilateral gonadectomy and salpingectomy. Pathological examination of the specimens revealed bilateral streak ovaries, with diagnoses of gonadoblastoma and dysgerminoma (Fig. 1E, F, H, and I). The patient was prescribed cyclic estrogen and progesterone replacement therapy.

On pathological examination, the left gonadal tumor measured 9.5 × 7.0 × 4.0 cm. The specimen was encapsulated, with a grayish-white color, and displayed multiple areas of calcification. The examined sections revealed characteristics of dysgerminoma, and the tumor cells tested positive for CD117 and SALL4. Additionally, foci of gonadoblastoma with hyalinization and calcification were also noted. The sections examined from the right streak gonad also exhibited features of gonadoblastoma. These findings confirmed our preoperative diagnosis.

### 2.2. Case 2

**Figure 2.**
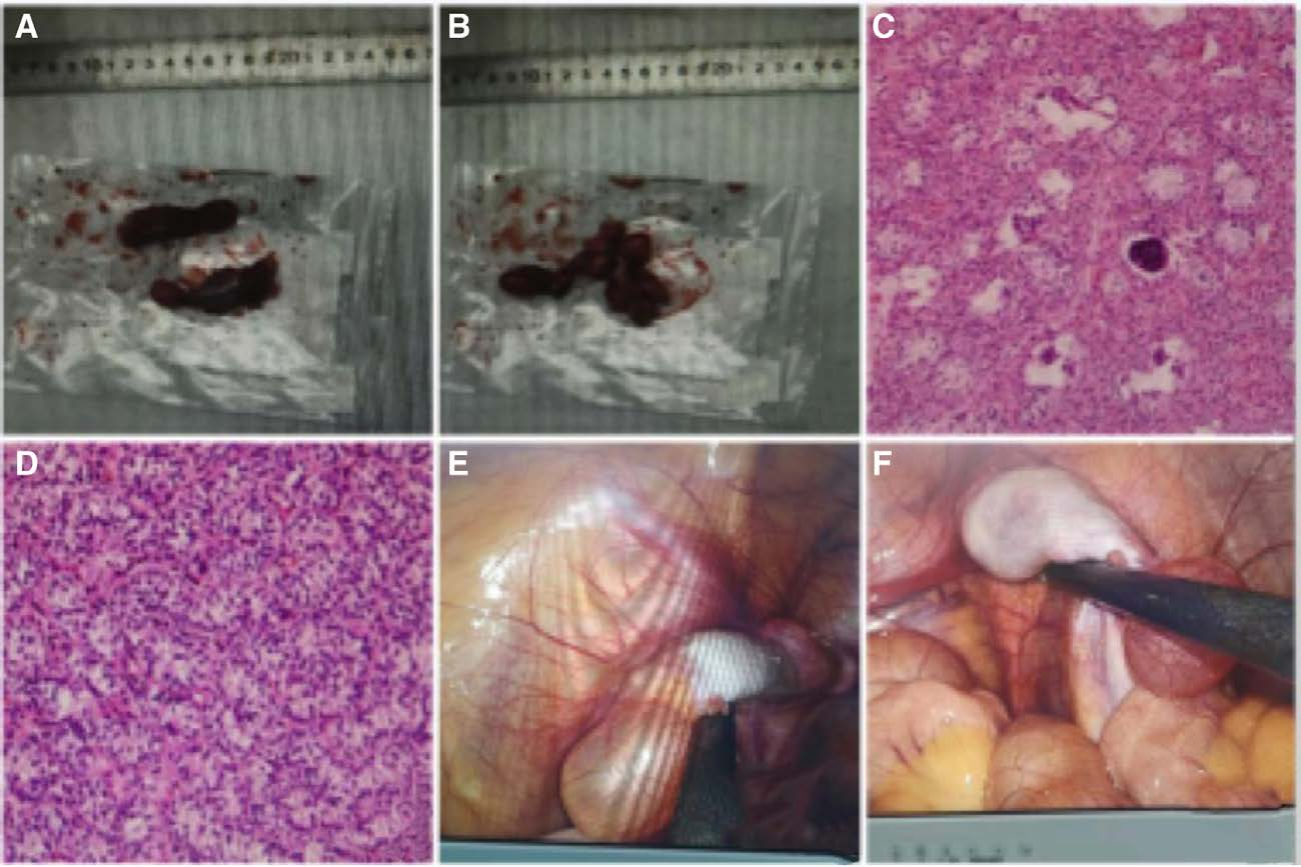
Clinical findings of a patient with XY complete androgen insensitivity syndrome. (A, B) Gross finding of inguinal testes in a patient with complete androgen insensitivity syndrome. (C, D) Microscopic finding of inguinal testes in a patient with complete androgen insensitivity syndrome, showing immature testicular tubules with a thick hyalinized basement membrane. (E, F) Bilateral adnexa.

A 16-year-old girl underwent evaluation for primary amenorrhea. Her weight was 60 kilograms, her height was 160 centimeters, and her body mass index was 23.4 kg/m². The patient exhibited no axillary hair, Tanner stage 2 breast development, and Tanner stage 1 pubic hair growth. Upon inspection, her external genitalia showed normal-appearing labia majora and minora. However, a shallow, blind vaginal pouch was detected with a depth of 3 cm. Ultrasonography and MRI revealed the absence of a uterus but nodular soft tissue shadows in both adnexa. Laboratory evaluation revealed follicle-stimulating hormone, luteinizing hormone, and estradiol serum levels of 15.51 IU/L, 33.25 IU/L, and 27.97 pg/mL, respectively. Serum testosterone levels were elevated at 4.62 ng/mL, and chromosomal analysis confirmed a normal male 46, XY karyotype. The patient was diagnosed with CAIS. Gonadectomy was recommended due to the potential for malignant transformation of occult testicular elements (Fig. 2A, B, E, and F). The surgery was uncomplicated, and the pathological examination of the gonads revealed Leydig cell hyperplasia and only Sertoli cells in the seminiferous tubules on both sides (Fig. 2C and D). The patient began estrogen therapy to promote breast development and improve bone health.

## 3. Discussion

46, XY pure gonadal dysgenesis was first described by Swyer in 1955 in 2 phenotypic women with 46, XY karyotype and unambiguously female external genitalia, primary amenorrhea and normal Müllerian structures. The incidence of this rare disease has been estimated at around 1 in 80,000 live births. 46, XY pure gonadal dysgenesis, can be caused by mutation in the sex-determining region on Y (SRY) gene located on the distal short arm of the Y chromosome (Yp11.3).^[5]^ Other mutation genes such as SOX9, DAX1, SF1, and WT-1 are involved in the regulation of SRY expression or act as a transcription activator downstream of SRY in the testis determining pathway that may also result in XY pure gonadal dysgenesis.^[9]^ The obstacle in testicular development result in the development of undifferentiated streak gonads, which cannot produce anti-Müllerian hormone or androgens.^[5]^ Because of the absence of androgen action, the vagina, cervix, uterus, and fallopian tubes develop, and external genitalia are those of females. Patients with CAIS (complete androgen insensitivity syndrome) have normal testes, and Leydig cells in the testicle produce testosterone. However, because of mutations in the androgen receptor gene, androgen action is deficient.^[5]^ The androgen receptor gene can be sequenced for any gene mutation by gene mutation analysis.^[10]^

Consequently, the Wolffan duct spontaneously regresses because of the absence of androgens and also cannot differentiate into the epididymis and vasdeferens. Furthermore, the Sertoli cells in the testes release anti-Müllerian hormone and induce the regression of Müllerian structures.^[5]^ Therefore, patients with CAIS have a short, blind end vagina without a uterus, ovaries, or fallopian tubes. The clinical treatment of the XY female patient generally includes prophylactic gonadectomy, appropriate hormone replacement therapy, and psychological counseling. Individuals whose karyotypes contain a Y cell are predisposed to gonadal ridge tumors, including gonadoblastomas, dysgerminomas, choriocarcinomas, and yolk sac tumors.^[11]^ The patients have a 30% probability of developing gonadoblastoma from dysgenetic gonads, while there is a 50% to 60% likelihood of malignant transformation of gonadoblastoma and, frequently to dysgerminoma. Prophylactic gonadectomy is recommended for these patients to prevent the risk of potential malignant changes of the gonads.^[11]^ In patients with XY pure gonadal dysgenesis, early gonadectomy is advised because of the high risk of malignant potential.^[5]^ In our case of XY pure gonadal dysgenesis, the patient underwent laparoscopic bilateral gonadectomy and salpingectomy soon after diagnosis, and can become pregnant through in vitro fertilization using donor oocytes.^[12]^ For girls with CAIS, the risk for testicular malignancy is lower than the adult hood,^[13]^ and a spontaneous puberty will occur including bone development and optimal breast. The testes remain produce levels of androgens that are enough to be converted to adequate estrogen levels for feminization. Exogenous HRT should be provided after gonadectomy, it is essential for XY female patients to initiate, mature, and maintain secondary sexual characteristics.^[14]^ Prevention of osteoporosis and coronary heart disease is benefit from the estrogen therapy.^[14]^ After the completion of estrogen-induced breast development, cyclic estrogen and progestogen therapy will prevent endometrial hyperplasia which may result by uncontrolled estrogen stimulation.^[14]^ For most patients with 46, XY have a completely female phenotype, female gender identity may be dominant. When difficulty for normal sexual satisfaction to penetration because of vaginal hypoplasia, traditional vaginoplasty, progressive vaginal dilation or laparoscopic Vecchietti may be considered.^[15]^ Moreover, patients with 46, XY DSD need proper counseling and education according to their psychosexual make-up and sociocultural factors. The work has limitations, and adding more cases could lead to a full understanding of the disease.

## 4. Conclusions

Laparoscopic bilateral prophylactic gonadectomy and long term hormone therapy with patient counseling and support is recommended.

## Author contributions

**Writing – original draft:** Tengge Yu.

**Writing – review & editing:** Tengge Yu, Li Liu.

**Data curation:** Li Liu.
